# Supplementary material for: Implementation measurement in global mental health: Results from a modified Delphi panel and investigator survey
Source: Glob Ment Health (Camb). 2023 Oct 31;10:e74. doi: 10.1017/gmh.2023.63 (PMC10663693; doi:10.1017/gmh.2023.63)
Supplement: Kemp et al. supplementary material [file S2054425123000638sup001.docx]

**Appendix: NIH RePORTER Search Strategy**

Projects funded since 1/1/2020

Text Search: ("mental health" OR "mental illness" OR "mental disorder" OR "depression" OR "anxiety" OR "ptsd" OR "alcohol abuse" OR "substance abuse" OR "schizophrenia" OR "psychosis")AND ("implementation science" OR "implementation research" OR "dissemination and implementation" OR "knowledge translation" OR "improvement science" OR "delivery science" OR "quality improvement")AND("Afghanistan" OR "Albania" OR "Algeria" OR "Samoa" OR "Angola" OR "Antigua" OR "Barbuda" OR "Argentina" OR "Armenia" OR "Azerbaijan" OR "Bangladesh" OR "Belarus" OR "Belize" OR "Benin" OR "Bhutan" OR "Bolivia" OR "Bosnia" OR "Herzegovina" OR "Botswana" OR "Brazil" OR "Bulgaria" OR "Burkina Faso" OR "Burundi" OR "Cambodia" OR "Cameroon" OR "Cabo Verde" OR "Central African Republic" OR "Chad" OR "Chile" OR "China" OR "Colombia" OR "Comoros" OR "Congo" OR "Costa Rica" OR "Côte d'Ivoire" OR "Cote d’Ivoire" OR "Ivory" OR "Cuba" OR "Djibouti" OR "Dominica" OR "Dominican" OR "Ecuador" OR "Egypt" OR "Salvador" OR "Eritrea" OR "Ethiopia" OR "Fiji" OR "Gabon" OR "Gambia" OR "Georgia" OR "Ghana" OR "Grenada" OR "Guatemala" OR "Guinea" OR "Guinea-Bissau" OR "Guyana" OR "Haiti" OR "Honduras" OR "India" OR "Indonesia" OR "Iran" OR "Iraq" OR "Jamaica" OR "Jordan" OR "Kazakhstan" OR "Kenya" OR "Kiribati" OR "Korea " OR "Kosovo" OR "Kyrgyz " OR "Lao" OR "Laos" OR "Latvia" OR "Lebanon" OR "Lesotho" OR "Liberia" OR "Libya" OR "Lithuania" OR "Macedonia" OR "Madagascar" OR "Malawi" OR "Malaysia" OR "Maldives" OR "Mali" OR "Marshall" OR "Mauritania" OR "Mauritius" OR "Mexico" OR "Micronesia" OR "Moldova" OR "Mongolia" OR "Montenegro" OR "Morocco" OR "Mozambique" OR "Myanmar" OR "Namibia" OR "Nepal" OR "Nicaragua" OR "Niger" OR "Nigeria" OR "Pakistan" OR "Palau" OR "Panama" OR "Papua New Guinea" OR "Paraguay" OR "Peru" OR "Philippines" OR "Romania" OR "Russia" OR "Russian" OR "Rwanda" OR "Samoa" OR "Sao Tome" OR "Senegal" OR "Serbia" OR "Seychelles" OR "Sierra Leone" OR "Solomon Islands" OR "Somalia" OR "South Africa" OR "Sri Lanka" OR "St. Lucia" OR "St. Vincent" OR "Grenadines" OR "Sudan" OR "Suriname" OR "Swaziland" OR "Syrian" OR "Syria" OR "Tajikistan" OR "Tanzania" OR "Thailand" OR "Timor-Leste" OR "Togo" OR "Tonga" OR "Tunisia" OR "Turkey" OR "Turkmenistan" OR "Tuvalu" OR "Uganda" OR "Ukraine" OR "Uruguay" OR "Uzbekistan" OR "Vanuatu" OR "Venezuela" OR "Vietnam" OR "West Bank" OR "Gaza" OR "Yemen" OR "Zambia" OR "Zimbabwe ") (Advanced); Search in: Projects AdminIC: All; Project Start Date:01/01/2010; Fiscal Year: All Fiscal Years

564 study results

467 excluding studies funded by VA, and D43s, R13, UG1, UL1

136 different contact-PIs

**Appendix: Full List of Expert Panel Members**

Pamela Collins (*withdrew*)

Professor of Psychiatry and Behavioral Sciences

Professor of Global Health

Director, Global Mental Health Program

University of Washington

Seattle, WA, USA

Shannon Dorsey

Adjunct Professor, Global Health

Professor, Psychology

University of Washington

Seattle, WA, USA

Usman Hamdani

Assistant Director and Associate Research Fellow

FaNs for Kids Project

Human Development Research Foundation

Islamabad, Pakistan

Michael Herce

Director, Implementation Science

Centre for Infectious Disease Research in Zambia (CIDRZ)

Lusaka, Zambia

Assistant Professor of Medicine, Division of Infectious Diseases

University of North Carolina

Chapel Hill, NC, USA

Brian Pence

Professor of Epidemiology

Associate Director, Division of Global Mental Health

Affiliate Faculty, Injury Prevention Research Center

University of North Carolina

Chapel Hill, NC, USA

Giuseppe Raviola

Director, Program in Global Mental Health and Social Medicine

Harvard Medical School

Assistant Professor of Psychiatry

Massachusetts General Hospital

Associate Director, The Chester M. Pierce, MD Global Psychiatry Division

Director, Mental Health

Partners In Health

Boston, MA, USA

Miguel Uribe

Medical Doctor and Psychiatrist

Associate Professor, Department of Social and Preventive Medicine

Pontificia Universidad Javeriana, School of Medicine

Bogotá, Colombia

Bryan Weiner

Professor, Global Health

Professor, Health Services

University of Washington

Seattle, WA, USA

**Appendix: Modified Delphi Results**

**Section 1: Measure Characteristics**

Question 1: Establishing characteristics in a new setting

Response options for Section 1 (Measure Characteristics)

1. I can rely on evidence of this characteristic from a different context (e.g. high-resource setting) and do not need to establish this characteristic in my setting
2. **I can rely on evidence of this characteristic from a similar context (e.g. low-resource setting) and do not need to establish this characteristic in my setting**
3. Ideally, I would assess this characteristic in my setting before proceeding
4. I must establish this characteristic in my setting before proceeding
5. It depends (e.g., on whether I am running a formal trial vs. conducting routine CQI activities, or on whether the measure is a key predictor/outcome in my study, or on the level of analysis, etc.). Please describe the circumstances under which would or would not need to establish this characteristic in your setting.
6. I do not know.

**Table X.1: Delphi Panel Judgements of the Transferability of Selected Implementation Measure Characteristics**

|  | | Most Frequent Response Option(s) |
| --- | --- | --- |
| *Validity* | | |
| Substantive content validity | | **2**  (n=3) |
| Discriminant content validity | | **2**  (n=4) |
| Predictive validity | | **2**  (n=3) |
| Concurrent validity | | **2**  (n=5) |
| Convergent validity | | **2**  (n=4) |
| Discriminant validity | | **2**  (n=4) |
| Known-groups differentiation | | **2**  (n=3) |
| Correlation analysis | | **2**  (n=5) |
| Cross-cultural validity | | **3**  and **4** (both n=2) |
| *Dimensionality* | | |
| Dimensionality | | **2** and **3** (both n=2) |
| *Reliability* | | |
| Internal consistency | | **2** and **3** (both n=2) |
| Test-rest reliability | | **2** (n=2) |
| *Pragmatic qualities* | | |
| Acceptable | **2** and **4** (n=2) | |
| Offers relative advantage over existing methods | **2** and **3** (n=2) | |
| Completed with ease | **2** (n=3) | |
| Compatible | **2** (n=3) | |
| Fits organizational activities | **4** (n=3) | |
| Informs clinical or organizational decision-making | **4** (n=2) | |
| Cost | **1** and **2** (n=2) | |
| Uses accessible language | **2** and **4** (n=2) | |
| Assessor burden (training) | **2** and **4** (n=2) | |
| Assessor burden (interpretation) | **2** (n=2) | |
| Length | **1** (n=2) | |

Question 2: Relative Importance of Pragmatic Measures

Respondents were asked to rate (on a scale of 1-4) how important each pragmatic characteristic would be when choosing among measures of equal validity/reliability/dimensionality.

**Table X.2: Delphi Panel Pragmatic Qualities Importance Ratings**

|  | Average Score |
| --- | --- |
| Acceptable (*Do users like the measure?*) | 3.8 |
| Completed with ease (*How hard is the measure to complete?*) | 3.8 |
| Cost (*Is the measure free to use?*) | 3.8 |
| Uses accessible language (*What is the reading level of the measure?*) | 3.8 |
| Appropriate (*Does use of the measure interfere with service implementation?*) | 3.4 |
| Length (*How many items does the measure have?*) | 3.2 |
| Informs clinical or organizational decision-making (*Are the measure findings actionable?*) | 3 |
| Fits organizational activities (*Does the measure map to actual services?*) | 2.8 |
| Assessor burden (training) (*How much training is required to learn how to administer the measure?*) | 2.8 |
| Assessor burden (interpretation) (*Does the measure have clear cut-offs, instructions for handling missing data, and generating summary scores?*) | 2.8 |
| Offers relative advantage over existing methods (*Is the measure better than other approaches to assessment of the same construct?*) | 2.4 |

**Section 2: Validation Strategies**

RESULTS: Table X.3 presents the average rating for each of the 8 dimensions for which analysis was possible. The degree of agreement across raters varied substantially by strategy.

**Table X.3: Average dimension scores by validation strategy type**

|  | Informal Elicitation | Formal Elicitation | Translation/Back-Trans | Survey w/ Measures | Survey w/ Outcomes | Vignettes | Evaluation  (n=3) |
| --- | --- | --- | --- | --- | --- | --- | --- |
| Confidence | 4.0 | 7.0 | 6.8 | 8.2 | 8.0 | 6.8 | 3.0 |
| Feasibility | 8.4 | 5.4 | 6.8 | 6.0 | 5.6 | 6.4 | 7.7 |
| Adaptability | 8.8 | 5.2 | 5.8 | 5.2 | 5.6 | 6.4 | 7.3 |
| Acceptability | 8.4 | 7.25 | 7.8 | 7.0 | 6.8 | 6.6 | 7.7 |
| Complexity | 2.8 | 5.0 | 6.0 | 6.0 | 8.0 | 5.2 | 4.3 |
| Time required | 3.6 | 5.6 | 6.6 | 7.0 | 8.4 | 5.8 | 4.7 |
| Expected cost | 2.2 | 4.2 | 5.4 | 6.0 | 7.2 | 5.0 | 4.3 |
| Compatibility | 8.4 | 5.6 | 8.8 | 6.4 | 6.2 | 7.2 | 7.0 |

Notes: Multiple respondents indicated they were unable to provide ratings for program evaluation because of the range of possible methods included in it.
